# Calculation of the disease burden associated with environmental chemical exposures: application of toxicological information in health economic estimation

**DOI:** 10.1186/s12940-017-0340-3

**Published:** 2017-12-05

**Authors:** Philippe Grandjean, Martine Bellanger

**Affiliations:** 1 Department of Environmental Health, Harvard T.H. Chan School of Public Health, Boston, MA USA; 2 University of Southern Denmark, Odense, Denmark; 3 EHESP School of Public Health, Paris, France

**Keywords:** Attributable risk, Burden of illness, Lead, Mercury, Neurotoxicity, Pesticides

## Abstract

Calculation of costs and the Burden of Disease (BoD) is useful in developing resource allocation and prioritization strategies in public and environmental health. While useful, the Disability-Adjusted Life Year (DALY) metric disregards subclinical dysfunctions, adheres to stringent causal criteria, and is hampered by gaps in environmental exposure data, especially from industrializing countries. For these reasons, a recently calculated environmental BoD of 5.18% of the total DALYs is likely underestimated. We combined and extended cost calculations for exposures to environmental chemicals, including neurotoxicants, air pollution, and endocrine disrupting chemicals, where sufficient data were available to determine dose-dependent adverse effects. Environmental exposure information allowed cost estimates for the U.S. and the EU, for OECD countries, though less comprehensive for industrializing countries. As a complement to these health economic estimations, we used attributable risk valuations from expert elicitations to as a third approach to assessing the environmental BoD. For comparison of the different estimates, we used country-specific monetary values of each DALY. The main limitation of DALY calculations is that they are available for few environmental chemicals and primarily based on mortality and impact and duration of clinical morbidity, while less serious conditions are mostly disregarded. Our economic estimates based on available exposure information and dose-response data on environmental risk factors need to be seen in conjunction with other assessments of the total cost for these environmental risk factors, as our estimate overlaps only slightly with the previously estimated environmental DALY costs and crude calculations relying on attributable risks for environmental risk factors. The three approaches complement one another and suggest that environmental chemical exposures contribute costs that may exceed 10% of the global domestic product and that current DALY calculations substantially underestimate the economic costs associated with preventable environmental risk factors. By including toxicological and epidemiological information and data on exposure distributions, more representative results can be obtained from utilizing health economic analyses of the adverse effects associated with environmental chemicals.

**Electronic supplementary material:**

The online version of this article (10.1186/s12940-017-0340-3) contains supplementary material, which is available to authorized users.

## Background

**Fig. 1:**
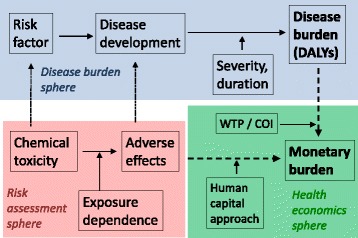
For individual environmental risks, the costs of exposure-dependent adverse effects can be estimated using health economics methods, and they can then be compared to costs associated with the estimated disease burden in terms of disability adjusted life years

To guide the most efficient use of limited resources for the purpose of reducing major origins of death and disability, researchers have attempted to estimate burdens of disease and catalogued them by risk factors. The first calculations of the global burden of disease were released in the World Development Report in 1993 [1]. The common metric most often used in measuring the Global Burden of Disease (GBD) is the Disability-Adjusted Life Year (DALY) [2], which combines duration and quality of life into a common metric that can be applied across diseases and organ systems (Fig. 1).

DALY calculations have proven highly useful for comparisons of the relative importance of major diseases and risk factors. Yet, the data available have so far allowed separation only of a limited number of risk factors and etiologies and have required merging some of them into clusters [3]. For example, the 2013 GBD report concluded that elevated blood pressure was the largest contributor to lost DALYs globally, while increased body mass index was the third largest contributor [3]. Both conditions likely represent intermediate steps in the pathogenesis of disease, and current evidence suggests that industrial chemicals may contribute to disease causation via hypertension and/or obesity [4–7].

An overall limiting factor is that calculations require decisions on causal associations, distributions of exposure to each risk factor in global populations and estimation of etiological effect sizes and disabilities [8]. The exact algorithm and the complex computations of the GBD also make it difficult to assess the validity of the summary estimates, which may cause disagreement [9]. Thus, apart from sources of infectious disease risks, the 2013 GBD report [3] estimated environmentally-attributable DALYs lost only for certain occupational hazards, ambient air pollution, household air pollution from solid fuel burning, residential exposure to radon, and childhood lead exposure. In total, the selected environmental risk factors contributed about 127 million DALYs or 5.18% of all DALYs lost [3]. Although substantial knowledge gaps remain, preventable environmental chemical exposures are known to include pesticides [10] and arsenic-contaminated drinking water [11] as major contributors to ill health on a global scale. However, these risks are not yet included in the GBD assessment. In addition, industrial chemicals can contribute to disease and dysfunctions, for example, by interfering with hormonal functions (endocrine disrupting chemicals, or EDCs) [12, 13]. Further, adverse effects on brain development due to lead, methylmercury and other neurotoxicants have been highlighted as major societal costs, even in the absence of diagnosed neuropsychiatric disease [14–16].

This review therefore seeks to identify approaches that can be used to complement the existing DALY calculations by assessing adverse health effects from available data on exposure levels and exposure-response relationships, while taking into account known uncertainties. We illustrate how health economic methods can be utilized to document the value of preventing subclinical functional changes that are not readily expressed in terms of DALYs. For these comparisons, we apply country-specific economic values of a DALY so that the toxicology-based economic estimates can be compared to DALY losses (Fig. 1).

### Toxicology-based health economics approach

Among the methods that may be applied to estimate the environmental burden of diseases (BoD), this article focuses on using assessments of environmental chemical exposures in combination with the economic value of environmentally-related adverse health outcomes. We then compare the economic estimates of health impacts that include subclinical functional changes for several major types of exposures with sufficient data for this kind of analysis. To the extent possible, these results are compared with the less extensive DALY estimates.

To ensure the widest possible coverage of our work, we searched the PubMed database for studies and reports published in English from 2000 to 2015 using terms including “cost” or “economic” in combination with the terms “environmental” or “chemical” and “impact” or “exposure.” We also searched cited references to identify additional relevant papers not retrieved by this search. In addition, we searched the websites of Eurostat, the Organization for Economic Cooperation and Development (OECD) and the World Health Organization (WHO).

### Attributing disease burdens to environmental exposures

As recommended by the Institute of Medicine, the BoD attributable to an environmental risk factor can be calculated as a product of three factors [17]:

$$ \mathrm{Disease}\  \mathrm{burden}=\mathrm{Disease}\  \mathrm{rate}\ \mathrm{x}\ \mathrm{AF}\ \mathrm{x}\ \mathrm{Population}\  \mathrm{size} $$

where AF is the Attributable Fraction (i.e., the percentage of a particular disease category that would be eliminated if the environmental risk factor were reduced to its lowest feasible level) [18]. The AF is the product of the prevalence of a risk factor multiplied by the relative risk of disease associated with that risk factor [19].

In considering exposure-outcome relationships, the GBD project relies upon expert input to select those links that meet stringent criteria for causality [3, 8]. A consequence of this strategy is that it favors established causal connections, for example, for infectious disease or injury, and those that have been supported by randomized clinical trials. In environmental health, intervention studies are often too complex or long-lasting to provide necessary documentation on the adverse impact of environmental risk factors [20]. Although the BoD calculations require knowledge on exposure-dependent outcomes and distributions of exposures, it would not be appropriate to interpret the absence of such information as support for an AF of zero [21, 22], although this is commonly given as the reason for not including such risk factors in BoD estimates. In contrast, the Intergovernmental Panel on Climate Change (IPCC) has prudently applied a weight-of-evidence characterization for probability of causation [23]. In dealing with thousands of potentially disease-causing chemicals, a realistic and precautionary approach would require characterization of the possible impact also of those substances that have not yet proven to cause adverse effects [21].

Expert elicitation methods have been developed to achieve consensus on AFs, while taking into account uncertainties regarding multifactorial causation and the impact of individual environmental chemicals [24, 25]. For such purposes, the Delphi method was developed on the premise that group judgments are more valid than those of individuals [26, 27]. Both this approach and expert elicitation have been applied by WHO to provide quantitative estimates on the fractions of 85 diseases attributable to the environment. The WHO authors considered that a total of 24% of the global BoD were reasonably due to modifiable environmental risk factors [28], with a more recent update resulting in a 22% contribution [29]. Although focusing mainly on specific diseases and less clearly on subclinical dysfunctions, the WHO estimate greatly exceeds the estimate of 5.18% in the GBD report [3].

Existing epidemiologic knowledge can be systematically evaluated in regard to causal attributions using the rigorous criteria developed by the GRADE Working Group [30, 31]. This approach was recently applied by the WHO in assessing disease causation by household fuel burning [32]. To assess the plausibility of a causal association, experimental toxicology information must similarly be systematically evaluated, and criteria for this purpose have been developed by the U.S. National Toxicology Program [33], the Danish Environmental Protection Agency [34], and several academic groups [35–37].

A recent project evaluated the impact of environmental exposures to EDCs within the EU [38], using the IPCC methodology for assessment of causation [23], the epidemiology criteria defined by the GRADE Working Group [30, 31], and the Danish Environmental Protection Agency classification of the strength of human and laboratory evidence for disease causation [34]. Similar calculations were subsequently carried out for the US [39], thereby contributing to a better coverage of global costs for the well-studied chemical risks.

### Monetizing the environmentally-related health outcomes

The economic estimates associated with the exposure-associated outcomes are typically calculated by the human capital approach, as based on the indirect costs [40, 41], i.e., the value of resources foregone and output lost due to illness, such as lost earnings, along with the estimated direct costs from medical treatment, as assessed by the Cost of Illness (CoI) method [42, 43]. The annual costs (or lifetime costs) are then estimated. The human capital approach provides important advantages, as it is transparent and allows assessment of costs associated also with regard to subclinical dysfunctions that may occur without necessarily being linked to a formal medical diagnosis and treatment.

Economic losses due to dysfunctions, such as cognitive deficits impacting productivity are generally calculated from projected life-time earnings converted to present-day value by discounting, while taking into account the Purchasing Power Parity (PPP) [44]. Using data relating intelligence quotient (IQ), labor force participation and schooling, the economic cost of a lost IQ point can be quantified through lost lifetime economic productivity [44, 45]. Overall, a cognitive skill improvement by one SD (i.e. 15 IQ points) is associated with 12% and 16% increases in annual labor earnings in high-income countries and low and middle-income countries (LMICs), respectively [46]. On the standard assumption that labor income represents half of the Gross National Income (GNI), as based on the World Bank World Development Indicators, the global economic loss associated with cognitive deficits can be derived as a percentage of the GNI [47].

Overall, we assume a societal perspective, where all costs borne by society are relevant (i.e., costs borne by the health care system, by the individual and the household, and by employers and insurers, as applicable). All estimates of economic costs are given in $(US)_2010_, with value range [in square brackets] from sensitivity analyses when available. Additional results are provided to show percentages of the global domestic product (GDP) or relative costs for given countries or regions studied, also for the year 2010.

To quantify and compare the costs of adverse health end points, the monetary value of a DALY or QALY (Quality-Adjusted life years) needs to be ascertained. While no official consensus of the appropriate value of a DALY/QALY has been reached, a conversion based on the Value of Life Year (VOLY) has been considered as the most reasonable choice [48, 49]. Different Economic values of DALYs have been allocated per income country group level, as defined by the World Bank. We chose the median VOLY value of $52,320 [$32,700 - $130,800], in terms of 2010 values adjusted for differences in per capita GINI 2010 at PPP and for inflation, as the basis for the DALY value estimates [50]. Each DALY value per income group level was adjusted based on the ratio of GNI per capita of the given group to the GNI per capita of the EU 27 as follows:

$$ DALY\;{UV}_{IC}= DALY{UV}_{EU}\frac{2010 ppp\;{CGL}_i\; GNI\; per\; capita}{2010 ppp\; EU\; GNI\; per\; capita} $$

with DALY*UV*_*ICGi*_= Unit value for a DALY in the EU; with *CGL*_*i*_ = country group level i GNI per capita and *i* = 1 to 5; *CGL*_1_ is for Low Income Country, *CGL*_2_ for Lower Middle Income Country; *CGL*_3_ is for Upper Middle Income Country and *CGL*_4_for High Income Country; and *CGL*_5_ includes all Low and Middle Income countries, since some environmental population attributable fractions were available at that income level and for High Income Countries.

Using the above dollar values, toxicology-based health economics calculations can now be compared with published DALY estimates (Fig. 1). Important groups of environmental chemicals of likely impact on the BoD include neurotoxicants that impact human brain development [16, 51], air pollution [52] and EDCs [12, 13]. Evidence on these substances is now reviewed to determine how future GBD calculations may more comprehensively consider BoD estimates that include wider ranges of dysfunctions and groups of chemicals.

### Estimates for major environmental risk factors

#### Lead and other neurotoxicants

The GBD report estimates the global burden due to lead poisoning in terms of intellectual disabilities (or mental retardation) [3], from which a global economic cost of $5 [$3.15–$12.6] billion per year can be derived (Table 1). However, this estimate does not consider IQ losses within the normal range and, therefore, fails to capture the societal losses for those children who are not shifted into the subnormal range of cognitive function. A more comprehensive assessment of children experiencing an IQ loss from childhood lead exposure in LMICs alone suggested an economic cost of $1.04 [$0.776 – $1.237] trillion from the sensitivity analysis [53], a value nearly 200-fold greater than the cost obtained from the GBD approach.

**Table 1:** Estimates of economic costs associated with lead and other neurotoxicant exposure

Risk factor	Adverse consequences	Context	Economic cost ($billions)	% GDP	% of Global GDP
Lead exposure	Cognitive deficits	LMICs [53]	1040 [775.5–1237]	5.20 [3.9–6.2]	1.68 [1.25–1.99]
		U.S. [57]	54.0 [47.5–64.3]	0.37 [0.33–0.44]	0.09 [0.08–0.1]
		EU [82]	60.6 [53.7–72.2]	0.36 [0.32–0.43]	0.1 [0.09–0.12]
		Total (sum)	1154 [876.7–1373.5]	2.47 [1.88–2.94]	1.83 [1.39–2.18]
	Intellectual disability only	World (WHO) [29]	16 [10–40]	<0.01	<0.01
		World (GDB) [3]	246 [154–615]	0.4 [0.24–1]	0.4 [0.24–1]
	Neurotoxicity total	World (WHO) [29]	5 [3.15–12.6]	<0.01	<0.01
		World (GBD) [3]	283 [177–708]	0.45 [0.27–1.1]	0.45 [0.27–1.1]
Methylmercury	Cognitive deficits	U.S. [58]	4·8 [4.2–5.7]	0.03 [0.026–0.04]	<0.01
		EU [56]	10.8 [9,6–11.2]	0.06 [0.053–0.062]	<0.01
		Sum	15.6 [13.8–16.9]	0.05 [0.044–0.54]	<0.01
Organophosphate pesticides	Cognitive deficits	U.S. [39]	44.7 [14.6–59.5]	0.30 [0.1–0.4]	0.07 [0.2–0.09]
		EU [69]	194 [62–259]	1.14 [0.37–1.52]	0.31 [0.09–0.4]
		Sum	248.7 [76.6–318.5]	0.8 [0.25–1.02]	0.38 [0.11–0.49]
Polybrominated diphenyl ethers	Cognitive deficits	U.S. [39]	266 [133–367]	1.8 [0.9–2.5]	0.4 [0.2–0.6]
		EU [69]	12·6 [2.08–29.4]	0.07 [0.011–0.16]	0.02 [0.003–0.05]
		Sum	278.6 [135.08–396.4]	0.9 [0.43–1.28]	0.42 [0.23–0.65]

An extended assessment suggests that the estimated costs of cognitive impairment associated with known childhood lead exposure represent about 1.83% [1.39% – 2.18%] of the global GDP in 2010 (Table 1), which is more than 4-fold greater than the similar value for DALYs valued by the GBD study (0.45%) [3] and by the WHO (0.4%) [29]. An earlier estimate that relied on extrapolation from US data suggested that the phase-out of lead in petrol has resulted in global benefits of $2.45 trillion per year [54]. When using a country-specific DALY value of $79,483 [49,677–198,798], this amount corresponds to approximately 30 [12.5–50] million DALYs, about three-fold higher than the estimate by the GBD authors. Thus, although lead was included as an environmental risk factor in the GBD report, the DALY numbers do not, by far, represent the total impact on human health by this global neurotoxicant.

Methylmercury is another neurotoxicant with detailed dose-response data and exposure information [55], but it has not been considered in the GBD report. Relying on recently updated dose-response data, annual costs for the EU [56] and the US [57, 58] have been estimated at a total of about $15.6 [$13.8 – $16.9] billion. As the distribution of methylmercury exposures varies substantially with dietary intakes of predatory fish [59–61], global costs are difficult to estimate at present.

Used as chemical flame retardants, the polybrominated diphenyl ethers (PBDEs) have been linked to IQ deficits in prospective studies of birth cohorts [62–64]. Organophosphate pesticides (OPs) may elicit similar deficits [65–67]. Because endocrine disruption is a possible mode of action for these substances [68], neurotoxicity was included in recent calculations of costs associated with EDC exposures [69]. In the EU, IQ losses due to PBDEs resulted in economic productivity losses of $12.6 [$2.8 – $29.4] billion, whereas OPs resulted in lost economic productivity of $194 [$62 – $259] billion. In the U.S., where exposures are different, similar data suggest losses of $266 [$133 – $367] billion and $44.7 [$14.6 – $59.5] billion, for PBDEs and OPs, respectively [39]. These costs thereby total almost $500 [$200 –$700] billion, a value that corresponds to about 10 million DALYs (i.e. close to the GBD estimate for lead alone [3]). These recent studies also calculated additional costs associated with exposure-related increases in the occurrence of ADHD and autism, but these costs are smaller and are therefore not considered here.

These four types of neurotoxicants contribute costs that represent more than 2.5% of the global GDP (Table 1) and belong to a larger group of twelve neurotoxic substances found to be convincingly associated with adverse effects on human brain development [15]. Although neurotoxic arsenic is a serious water contaminant in many countries [11, 70], insufficient evidence is available at this time to calculate costs. Even so, arsenic costs may well be of a magnitude similar to the one associated with lead exposure. Thus, for most of the other known neurotoxicants, plausible cost estimates cannot be produced at present due to insufficient exposure documentation and in some cases uncertain dose-response relationships, especially at low exposure levels.

An important reason that DALY calculations for neurotoxicants are seriously underestimated is that they reflect costs only for intellectual disabilities, and solely those attributed to lead exposure [3]. For comparison, OP exposure is estimated to cause 59,300 cases of intellectual disability in the EU and 7500 cases in the U.S. [39, 69]. In fact, when compared to the costs linked by the GBD to intellectual disabilities alone, the costs that we estimate from OP-associated cognitive dysfunction were six fold higher in the EU [69] and three-fold higher in the U.S. [39].

Optimal brain functions, not just the absence of intellectual disabilities, are necessary for health and for productivity in society, and the brain therefore differs from most other organ systems, for which minor decrements may be fully compatible with health [16]. Thus, focusing only on intellectual disabilities and disregarding less severe cognitive dysfunction results in a substantial underestimation of the total societal costs due to neurotoxicity.

### Air pollution

In regard to ambient air pollution as an environmental health risk factor, the GBD calculations [3] reported a total of 74.7 million DALYs lost due to particulate matter and ozone leading to a crude estimate of $1.1 [0.677–2.7] trillion in annual costs (Table 2). For comparison, WHO estimates for particulate matter show 85 million DALYs lost, corresponding to $1.2 [0.74–2.9] trillion [29]. In both studies, the focus was on pulmonary disease, lung cancer, and cardiovascular effects.

**Table 2:** Estimates of economic costs associated with air pollution

Adverse consequence	Context	Economic cost ($billions)	% GDP	% of Global GDP
Asthma	U.S. [57]	2.33 [0.728–2.5]	0.02 [0.006–0.021]	<0.01
	EU [82]	1.70 [0.568–1.98]	0.01 [0.003–0.012]	<0.01
	EU city children [97]	0.151 [0.03–0.3]^a^	<0.01	<0.01
Preterm birth	U.S. [98]	4.3 [2.06–8.22]	<0.01	<0.01
Cardiovascular	EU [99]	37.24 [24.47–49.83]^a^	0.22 [0.14–0.29]	0.06
All health impacts	OECD countries [100]	500 [300–1250]	1.2 [0.7–2.8]	0.8 [0.5–2]
	China [100]	483 [300–1200]	8 [5–20]	0.8 [0.5–2]
	India [100]	120 [74–300]	7 [4–17]	0.2 [0.1–5]
	Sum (OECD, China, India)	1100 [700–2760]	2.2 [1.3–5.4]	1.8 [1.1–4.4]
	World (WHO) [52]	1177 [736–2942]	1.9 [1.1–4.6]	1.9 [1.1–4.6]
	World (GBD) [3]	1083 [677–2709]	1.7 [1.1–4.3]	1.7 [1.1–4.3]

Base case estimates are presented along with range [low/high end estimates] from sensitivity analysis or 95 CI^a^. All estimates are given in $2010, a 1.33 rate change € /$ is used, and for estimates prior to 2010, inflated adjustments are made. OECD estimates are based on DALYs reported for OECD countries (High Income Countries, HICs), China (Upper Middle Income Country, UMIC), & India (Lower Middle Income Country, LMIC). Our estimates of DALYs for OECD, WHO and GBD are based on Value of Life Year (VOLY) [50] adjusted to $2010 PPP and for inflation and then adjusted per income group levels from World Bank GNI per capita in $ppp2010. For consistency we valued OECD DALY estimates based on VOLY instead of using Value of Statistical Life Year (VSL) for mortality costs (additional 10% for morbidity included) as reported in OECD [100]

However, the GBD report [3] ignored several important outcomes. For example, longitudinal studies of particulate matter less than 2.5 μm in diameter have linked exposures during pregnancy to increased incidence of preterm birth or low birth weight [71]. Such outcomes are associated with direct medical care costs for the neonate, as well as long-term costs associated both with medical care and as a result of lower IQ.

Likewise, cognitive deficits have been linked to local air pollution both prenatally [72] and at school age [73]. Attribution to specific substances remains somewhat uncertain due to heterogeneities in the origins of these conditions and the complex compositions of air pollution as well as inconsistencies in study designs that also contribute to different findings in available studies [74]. Nonetheless, findings from toxicological studies add biological plausibility and credence to the results from observational studies [74–76]. Although this evidence has only recently been systematically evaluated [77], the cost estimates suggest that air pollution likely contributes much more to the environmental disease burden than summarized by the GBD report [3].

Asbestos may also be considered an ambient air pollutant [78, 79], although the GBD authors regarded it as an occupational risk factor only. Asbestos-associated cancers of the lung, mesothelium and other sites are likely underestimated for populations in general, and this may also be true for other adverse outcomes, especially in LMICs [80]. Other substances listed by the GBD report as occupational hazards may well contribute to community risks as well.

### Endocrine disruptors and other environmental hazards

International working groups have assessed environmental chemicals that are thought to cause disruption of endocrine functions, thus leading to a variety of diseases and dysfunctions [38, 81]. The experts used a modified Delphi approach as described above to deal with uncertainties and to estimate minimal societal costs. Although the toxic mechanism for some chemicals classified as EDCs may be unknown and may not necessarily reflect interference with hormonal functions, the working groups scrutinized exposures to EDCs in regard to relevant adverse outcomes. In addition to cognitive dysfunction, consensus was achieved for probable (>20%) attribution of chemical exposures in regard to childhood and adult obesity, testicular cancer, male infertility and mortality associated with reduced testosterone, fibroids, and endometriosis [81] – all conditions that are not usually considered in BoD calculations. Apart from cognitive deficits, the major estimated costs were associated with obesity, with one of the phthalates contributing most of the costs ($20.8 billion) [5].

The total costs of EDC exposures in the EU and the US have been estimated to be $217 [$110 - $359] billion [81] and $340 [$0.67 - $612] billion [39], respectively. Converted to DALYs the total costs represent 4.1[2.1–6.9] million DALYs for the EU countries and 4.3 [0.84–7.7] million DALYs for the US. These numbers constitute a minimum, as calculations were carried out only for substances and outcomes with a high probability of causation and for which exposure data were available. Again, these findings indicate that current estimates of the environmental BoD are much too low.

Economic impacts have been published for a variety of additional chemical exposures, most of which are not yet covered in the GBD (see Additional file 1: Table S1). However, most calculations identified are based on a variety of assumptions and cover different age strata and populations and therefore do not represent a systematic approach that would be required for calculation of global burdens of disease. Yet, the calculations illustrate the substantial contribution associated with adverse health outcomes and environmental etiologies not currently considered in the GBD estimates.

Diseases for which environmental cost estimates exist but are not covered by the 2013 GBD report include childhood asthma [57, 82], preterm birth [83], testicular cancer [84], male factor infertility [84], autism and ADHD [69], cryptorchidism [84], liver and lung cancer and toxicity [85], renal toxicity [85], childhood and adult obesity [5, 86], adult diabetes [5], fibroids and endometriosis [38], and coronary heart disease of chemical origin [84, 86].

In addition, important chemical exposures that are known to be associated with adverse health effects include a much broader array of industrial chemicals, such as aldrin [85], bisphenols [5], dichlorodiphenyltrichloroethane (DDE) [5, 38], lindane [85], organic and inorganic mercury [56, 57, 85], OPs [69], PBDEs [84] and phthalates [5, 69, 84, 87]. Further, risks associated with exposures to substances like arsenic and cadmium were considered in the 2013 GBD report [3] only as occupational, although they also occur in the general environment [7].

As a complement, and in the absence of sufficient evidence to generate overall chemical-related cost estimates, we used the environmental AFs judged by WHO experts for a range of relevant diagnoses [29] to calculate approximate DALY burdens and economic costs (Table 4). We note that several groups of diseases of major public health significance were judged to cause very substantial costs in terms of DALYs and economic expenses. The total annual cost for the groups of diseases selected by the WHO experts was about $4 [2.5–10.1] trillion, or 260 million DALYs. Although this DALY estimate is twice as high as the GBD calculation of 127 million for the environmental BoD [3], the estimates provided by WHO overlap only to a small extent with the risk factors considered by the GBD authors. Accordingly, the total environmental BoD would likely be substantially greater than calculated by either group. Further, our above compilation of costs due to neurotoxic chemicals (Table 1) is substantially higher than both the GBD estimate for lead exposure and the WHO calculation for neurobehavioral deficits, and our toxicology-based results therefore add further to the total costs.

### Implications

Our findings suggest that a revised paradigm is required for evaluating and prioritizing the environmental contribution to human illness and the associated costs. As an important requirement for proper assessment of the environmental BoD, lack of complete documentation should not be misconstrued to mean that an environmental risk factor has no adverse impact on health [21, 22]. A revised paradigm will have to use systematic, though less restricted, criteria for causal attribution, as already recommended by WHO [31]. While this has been achieved on regional scales in recent studies of EDCs [38, 39] and methylmercury neurotoxicity [56], obstacles are likely to occur in generating such analyses on a global scale due to paucity of exposure data and difficulties in estimating costs in different settings. Our approach utilizes both toxicology information and health economics methods and thereby represents an important supplement to currently used methods that result in serious underestimations.

At present, the outcomes and exposures covered by the literature represent only a small part of the chemical universe and their full spectrum of effects on human health. Still, the cost estimates add up to sizable amounts that is similar to the 5.18% of the narrowly defined environmentally-attributable DALYs reported in the 2013 GBD report [3]. The estimates provided by WHO from expert opinion suggest that environmental risks contribute approximately 260 million DALYs or twice as much as calculated by the GBD authors [29]. Our results are in reasonable agreement with some of WHO estimates, although the WHO-calculated costs for neurotoxicity (Table 4) are clearly much too low (Table 1).

Accordingly, the total cost estimated for specific risk factors with known toxicology and exposure data were evaluated at 5.3% of the global GDP (Tables 1, 2 and 3). Further, the 6.5% estimate from the AFs was obtained by WHO (Table 4), and the DALY calculations obtained by the GDB authors correspond to about half as much. As the risk factors considered by the three different approaches only partially overlap, the total environmental BoD costs likely exceed 10% of the global GDP.

**Table 3:** Estimates of economic costs associated with EDC exposures in the EU [81] and the US [39]

EDC	Adverse consequences	Context	Economic costs ($millions)	% GDP (2010)	% of Global GDP
Polybrominated diphenyl ethers (PBDEs)	Testicular cancer	US	81.5 [24.8–109.3]	<0.01	<0.01
		EU	1100 [416–1100]	<0.01	<0.01
	Cryptorchidism	US	35.7 [NA - NA]	<0.01	<0.01
		EU	172.6 [155.5–172.6]	<0.01	<0.01
Dichlorodiphenyl trichloroethane (DDE)	Childhood obesity	US	29.6 [NA - 57.3]	<0.01	<0.01
		EU	32.7 [NA - 114.8]	<0.01	<0.01
	Adult diabetes	US	1800 [NA – 13,500]	<0.01 [NA - 0.08]	<0.01
		EU	1100 [NA – 22,065]	<0.01 [NA - 0.13]	<0.01
	Fibroids	US	259 [NA - NA]	<0.01	<0.01
		EU	216.8 [NA - NA]	<0.01	<0.01
Di-2-ethylhexyl phthalate	Adult obesity	US	1700 [NA - NA]	0.011	<0.01
		EU	20,800 [NA - NA]	0.12	<0.01
	Adult diabetes	US	91.4 [NA - NA]	<0.01	<0.01
		EU	807.2 [NA - NA]	<0.01	<0.01
	Endometriosis	US	47,000 [NA - NA]	0.32	<0.01
		EU	1700 [NA - NA]	0.01	<0.01
Bisphenol A	Childhood obesity	US	2400 [NA - NA]	0.02	<0.01
		EU	2000 [NA - NA]	0.02	<0.01
Benzyphtalates & butylphalates	Male infertility resulting in Increasesed ART	US	2500 [NA - NA]	0.02	<0.01
		EU	6300 [NA - NA]	0.04	0.01
Phtalates	Low testoterone and increased early mortality	US	8800 [NA - NA]	0.06	0.012
		EU	10,600 [NA - NA]	0.05	0.012
Multiple exposures	Attention deficit hyperactivity disorder (ADHD)	US	698 [568–1950]	<0.01 [<0.01–0.011]	<0.01
		EU	3056 [1600–3800]	0.014 [<0.01–0.017]	<0.01
	Autism	US	1984 [803–4100]	0.014 [<0.01–0.024]	<0.01
		EU	352 [105–530]	<0.01	<0.01
All compounds included		US	340,000 [668–612,000]	2.33 [<0.01–3.53]	0.54 [<0.01 0.96]
		EU	217,000 [110,049–359,239]	1.2 [0.75–2.12]	0.34 [0.17–0.57]
		Sum	557,000 [110,707–971,239]	1.8 [0.3–3.07]	0.88 [0.17–1.54]

NA: Not available

Base case estimates are presented along with ranges [Low end and High end estimates] from sensitivity analyses, when available

All estimates are given in $2010, for EU a 1.33 rate change € /$ is used, and for estimates prior to 2010, inflated adjustments are made

**Table 4:** Environmental burdens of disease in terms of DALYs, economic value, and percent of GDP for major health outcomes based on attributable risks derived by WHO [29]^a^

Disease	DALYs (% fraction of total burden of disease in DALYs)	Environmental AF (%)	DALYs due to environmental risk factors	Economic cost ($billions)	% of Global GDP
*Respiratory diseases*					
Asthma	25,202,418 (0.9)	44 (26–53)	11,055,150	200 [124–500]	0.3 [0.2–0.8]
Chronic obstructive pulmonary disease	92,376,604 (3.4)	35 (20–48)	32,280,160	400 [250–1000]	0.6 [0.4–1.5]
Lower respiratory infections	146,863,685 (5.4)	35 (27–41)	51,752,605	530 [330–1320]	0.8 [0.5–2.1]
*Cancers*					
Lung cancer	38,535,303 (1.4)	36 (17–52)	13,902,105	285 [180–720]	0.5 [0.3–1.1]
Other cancers	185,421,704 (6.8)	16 (7–41)	31,047,781	715 [450–1800]	1.1 [0.7–2.8]
*Cardiovascular diseases*					
Ischemic heart diseases	165,717,210 (6.0)	35 (26–46)	58,561,915	1065 [670–2660]	1.8 [1.1–4.2]
Stroke	141,348,082 (5.2)	42 (24–53)	58,985,984	843 [527–2108]	1.3 [0.8–3.3]
*Neuropsychiatric diseases*					
Childhood behavioral disorders	6,208,771 (0.22)	12 (3–36)	742,156	12 [8–30]	0.02 [0.01–0.05]]]
*Total*			258,327,866	4050 [2500–10,140]	6.5 [4–15.8]

^a^Total DALYs, 2,743,857,491 (2012 values); Breakdown of DALYS for LMICs and HICs from WHO supplement [29]

Our calculations in terms of the global GDP suggest that several environmental risk factors represent very substantial annual losses. The application of different country-based unit values of DALYs generated detailed global cost estimates as well as value ranges, although caution is necessary when interpreting specific results. Our findings emphasize the need to consider updated and more comprehensive benefit-cost ratios when applying cost-effectiveness thresholds commonly used in economic evaluations in support of health priority setting or interventions [88, 89]. The data presented here suggest that environmental chemicals need to be more highly prioritized.

Of the close to 127 million DALYs attributed by the GBD report to environmental risks, ambient air pollution is a main contributor [3]. While this estimate is in approximate accordance with other calculations, adverse effects of air pollutants, e.g., on preterm birth and on brain development were not included. Lead exposure is also a major contributor due to the association with intellectual disabilities, but the total costs are much higher than the costs linked to intellectual disabilities only. Similarly, other developmental neurotoxicants, such as pesticides, methylmercury, and arsenic, not included in the GBD, are associated with costs that are at least as large as those caused by lead alone. Neurodevelopmental toxicity therefore must be considered a much greater contributor to the environmental BoD than indicated by the DALY losses. Our results therefore also suggest changes in the focus on environmental health strategies to protect human health.

While the GBD considered a high body mass index to be as important a risk factor as all environmental risk factors combined [3], exposures to phthalates and other endocrine disruptors represent a substantial attributable fraction for obesity [5, 90]. Some degree of environmental causation therefore seems to contribute to high body mass index as a main risk factor. The same is probably true for elevated blood pressure [7, 91]. Future calculations should therefore focus on environmental etiologies, rather than intermediate stages of the pathogenesis.

A major obstacle in assessing attributable risks from environmental chemicals is the incomplete documentation of causal associations and exposure distributions. Fortunately, techniques are available to overcome these difficulties [11, 38] so that more representative costs can be estimated from at least partial documentation, thereby avoiding the erroneous assumption of zero costs when the evidence is uncertain [22]. Individual levels of chemical exposures can often be measured by means of exposure biomarkers [92], but the current coverage is patchy, both in regard to substances and populations. Further, data that represent industrial chemical exposures outside of Europe and the U.S. are available only from a small number of countries.

Obtaining better estimates of exposure levels beyond Europe and North America is a necessary contribution to the achievement of the Sustainable Development Goals. For example, hazardous waste sites already constitute a main source of exposure to toxic agents in LMICs [93, 94]. The OECD estimates that, by 2030, the LMICs will comprise the leading sites for chemical manufacture and use [95], while infrastructures to protect public health and the environment may be insufficient [96]. Simple extrapolation from the American or European experience to estimate global attributable burdens would then be inappropriate. In addition, research must continue to better elaborate the effects of industrial chemicals in LMICs with weaker regulatory infrastructure to prevent uncontrolled exposures to vulnerable population groups, and where greater effects may ensue than those identified in industrialized countries. As a further consideration, the recent estimate of global costs of childhood lead exposure assumes that current PPP data could correct for differences in lifetime economic productivity, and the impact of IQ on economic productivity [53]. Given the much higher rates of growth in GDP per capita in some LMICs, especially in China, India and several countries of Southeast Asia, annual productivity gains are almost certainly higher than in industrialized countries. Consequently, PPP correction yields an underestimate of the true cost of childhood exposure to lead and other neurotoxicants. Given that LMICs vary in their health care delivery systems, estimating direct costs of medical care due to conditions that result from industrial chemical hazards cannot rely on simple extrapolation from a single country’s experience to an entire continent. Still, these uncertainties should not prevent prudent judgment and proper evaluation of the costs to society due to the environmental BoD.

The human capital approach that we used enabled the estimation of indirect costs, such as lost lifetime earnings, along with direct costs of medical treatment [42, 43]. Yet intangible costs, such as the value to an individual avoiding pain and suffering, may also be substantial [91]. Willingness To Pay (WTP) methods have been developed to capture these values. Thus, DALY values imply a WTP for disability prevention. Insofar as great care is taken to avoid double counting, WTP and COI methods can be complementary and may be leveraged to better quantify the complete societal value of prevention (Fig. 1).

The importance of these issues goes beyond the research community and health care institutions. Decisions made by health ministries regarding prevention are generally separate from the investments needed to prevent chronic conditions as by-products of industrial chemical exposures. Thus, health protection and health care decisions are usually taken without regard to necessary environmental regulation or modification in manufacturing, energy production, transportation, and other practices. When policy makers decide whether to limit effects of industrial chemical exposures, the narrow costs to industry are often presented as reasons not to proceed with protections to human health [96]. Concerns about costs to the private sector can appear particularly acute in the industrializing world context, where added emphasis is placed on accelerated economic growth that can induce further investments in health and other societal priorities. The present study illustrates that the current DALY approach is insufficient to estimate the total environmental BoD that would be of importance in generating useful guidance for policy decisions. Our results illustrate that a more comprehensive assessment of costs, also for dysfunctions and other outcomes less serious than mortality and diagnosed morbidity, is both necessary and feasible.

For proper evaluation of the environmental BoD, a new paradigm is needed to better inform decisions by clinicians, public health officials and regulatory agencies about the likely scope of disease and dysfunction associated with industrial chemicals. Recent studies have demonstrated that major obstacles in assessing attributable risks from environmental chemicals, such as the incomplete documentation of causal associations and exposure distributions, can be overcome [29, 38, 56]. Hence, while uncertainties in causation and distribution of environmental exposures will remain, they should not prevent realistic calculations of estimated disease burdens due to environmental risks. Our toxicology-economics approach can add to the proper recognition of preventable risk factors in both rich and poor countries alike as a necessary contribution to the achievement of the Sustainable Development Goals.

## Conclusions

The most recent assessment of Disability-Adjusted Life Year (DALY) losses estimated that environmental causation contributes only 5.18% of the total disease burden. However, these estimates ignore risks that are considered uncertain and exclude subclinical conditions, although the costs are certainly not zero. We relied on health economics methods to estimate societal costs associated with adverse outcomes of exposures to environmental chemicals. We highlight substances such as mercury, pesticides, brominated diethyl ethers, and several endocrine disrupting chemicals as serious health hazards that need to be confronted. Our results show that functional deficits, especially regarding cognition, greatly add to the total environmental Burden of Disease (BoD) and that total costs are substantially higher than those calculated in terms of the DALY losses that are linked to specific medical diagnoses. We also emphasize that environmental BoD assessments are easily underestimated, especially when focusing only on risk factors with detailed documentation, and when ignoring adverse effects beyond specific disease risks and mortality. Calculations to derive exposure-related health costs for comparison with DALY estimates should be encouraged to obtain more comprehensive and valid conclusions on the environmental BoD. Achievement of the Sustainable Development Goals will require this recognition of environmental risk factors as major contributors to human dysfunction, disease, and mortality in both rich and poor countries alike.

## Additional file

Additional file 1: Additional calculations of economic costs due to environmental disease burdens [101–105]. (DOC 39 kb)

## Abbreviations

ADHD: Attention Deficit Hyperactivity Disorder
AF: Attributable Fraction
ART: Assisted reproductive technology
BoD: Burden of Disease
COI: Cost of Illness
DALY: Disability-Adjusted Life Year
EDC: Endocrine Disrupting Chemical
GBD: Global Burden of Disease
GDP: Gross Domestic Product
GNI: Gross National Income
HIC: High Income Country
IPCC: Intergovernmental Panel on Climate Change
LMIC: Low and middle-income country, and Lower middle income country
OECD: Organization for Economic Cooperation and Development
PBDE: Polybrominated diphenyl ether
PPP: Purchasing Power Parity
QALY: Quality-Adjusted life years
UMIC: Upper Middle Income Country
VOLY: Value of Life Year
WHO: World Health Organization
WTP: Willingness to pay

## References

[1] World Bank (1993). World development report 1993. Investing in health.

[2] Zeckhauser R, Shepard D. Where now for saving lives? Law and Contemporary Problems. 1976:5–45.

[3] Risk Factors Collaborators GBD, Forouzanfar MH, Alexander L, Anderson HR, Bachman VF, Biryukov S, Brauer M, Burnett R, Casey D, Coates MM, Cohen A, Delwiche K, Estep K, Frostad JJ, Astha KC, Kyu HH, Moradi-Lakeh M, Ng M, Slepak EL, Thomas BA, Wagner J, Aasvang GM, Abbafati C, Abbasoglu Ozgoren A, Abd-Allah F, Abera SF, Aboyans V, Abraham B, Abraham JP, Abubakar I (2015). Global, regional, and national comparative risk assessment of 79 behavioural, environmental and occupational, and metabolic risks or clusters of risks in 188 countries, 1990-2013: a systematic analysis for the global burden of disease study 2013. Lancet.

[4] Heindel JJ, Newbold R, Schug TT (2015). Endocrine disruptors and obesity. Nat Rev Endocrinol.

[5] Legler J, Fletcher T, Govarts E, Porta M, Blumberg B, Heindel JJ, Trasande L (2015). Obesity, diabetes, and associated costs of exposure to endocrine-disrupting chemicals in the European union. J Clin Endocrinol Metab.

[6] Norman RE, Carpenter DO, Scott J, Brune MN, Sly PD (2013). Environmental exposures: an underrecognized contribution to noncommunicable diseases. Rev Environ Health.

[7] Cosselman KE, Navas-Acien A, Kaufman JD (2015). Environmental factors in cardiovascular disease. Nat Rev Cardiol.

[8] Lim SS, Vos T, Flaxman AD, Danaei G, Shibuya K, Adair-Rohani H, Amann M, Anderson HR, Andrews KG, Aryee M, Atkinson C, Bacchus LJ, Bahalim AN, Balakrishnan K, Balmes J, Barker-Collo S, Baxter A, Bell ML, Blore JD, Blyth F, Bonner C, Borges G, Bourne R, Boussinesq M, Brauer M, Brooks P, Bruce NG, Brunekreef B, Bryan-Hancock C, Bucello C (2012). A comparative risk assessment of burden of disease and injury attributable to 67 risk factors and risk factor clusters in 21 regions, 1990-2010: a systematic analysis for the global burden of disease study 2010. Lancet.

[9] Cohen J (2012). Health metrics. A controversial close-up of humanity's health. Science.

[10] Bourget D, Guillemaud T (2016). The hidden and external costs of pesticide use. Sustainable Agric Rev.

[11] Pruss-Ustun A, Vickers C, Haefliger P, Bertollini R (2011). Knowns and unknowns on burden of disease due to chemicals: a systematic review. Environ Health.

[12] Gore AC, Chappell VA, Fenton SE, Flaws JA, Nadal A, Prins GS, Toppari J, Zoeller RT (2015). EDC-2: the Endocrine Society's second scientific statement on endocrine-disrupting chemicals. Endocr Rev.

[13] Bergman A, Heindel JJ, Jobling S, Kidd KA, Zoeller RT: State of the science of endocrine disrupting chemicals 2012. In.: united National Environment Programme and world health Organization; 2013.

[14] Grandjean P, Landrigan PJ (2006). Developmental neurotoxicity of industrial chemicals. Lancet.

[15] Grandjean P, Landrigan PJ (2014). Neurobehavioural effects of developmental toxicity. Lancet Neurol.

[16] Grandjean P (2013). Only one chance. How environmental pollution Iimpairs brain development – and how to protect the brains of the next generation.

[17] Institute of Medicine (1981). Costs of environment-related health effects.

[18] Smith KR, Corvalan CF, Kjellstrom T (1999). How much global ill health is attributable to environmental factors?. Epidemiology.

[19] Hanley JA (2001). A heuristic approach to the formulas for population attributable fraction. J Epidemiol Community Health.

[20] Allen RW, Barn PK, Lanphear BP (2015). Randomized controlled trials in environmental health research: unethical or underutilized?. PLoS Med.

[21] Grandjean P, Gee D, Grandjean P, Hansen SF, van den Hove S, MacGarvin M, Martin J, Nielsen G, Quist D, Stanners D, II v (2013). Science for precautionary decision-making. Late lessons from early warnings.

[22] Gwinn MR, Axelrad DA, Bahadori T, Bussard D, Cascio WE, Deener K, Dix D, Thomas RS, Kavlock RJ, Burke TA (2017). Chemical risk assessment: traditional vs public health perspectives. Am J Public Health.

[23] Intergovernmental Panel on Climate Change: Guidance notes for lead authors of the IPCC Fourth Assessment Report on addressing uncertainties. 2005.

[24] Hald T, Aspinall W, Devleesschauwer B, Cooke R, Corrigan T, Havelaar AH, Gibb HJ, Torgerson PR, Kirk MD, Angulo FJ, Lake RJ, Speybroeck N, Hoffmann S (2016). World Health Organization estimates of the relative contributions of food to the burden of disease due to selected foodborne hazards: a structured expert elicitation. PLoS One.

[25] Knol AB, Slottje P, van der Sluijs JP, Lebret E (2010). The use of expert elicitation in environmental health impact assessment: a seven step procedure. Environ Health.

[26] Juri P (1971). The Delphi method: substance, context, a critique and an annotated bibliography. Socio Econ Plan Sci.

[27] Rescher N (1998). Predicting the future.

[28] Pruss-Ustun A, Bonjour S, Corvalan C (2008). The impact of the environment on health by country: a meta-synthesis. Environ Health.

[29] Pruss-Ustun A, Wolf J, Corvalan C, Neville T, Bos R, Neira M. Diseases due to unhealthy environments: an updated estimate of the global burden of disease attributable to environmental determinants of health. J Public Health (Oxf). 2017;39:464–475. 10.1093/pubmed/fdw085 PMC5939845 27621336

[30] Atkins D, Best D, Briss PA, Eccles M, Falck-Ytter Y, Flottorp S, Guyatt GH, Harbour RT, Haugh MC, Henry D, Hill S, Jaeschke R, Leng G, Liberati A, Magrini N, Mason J, Middleton P, Mrukowicz J, O'connell D, Oxman AD, Phillips B, Schunemann HJ, Edejer T, Varonen H, Vist GE, Williams JWJ, Zaza S (2004). Grading quality of evidence and strength of recommendations. BMJ.

[31] Schunemann HJ, Oxman AD, Brozek J, Glasziou P, Jaeschke R, Vist GE, Williams JWJ, Kunz R, Craig J, Montori VM, Bossuyt P, Guyatt GH (2008). Grading quality of evidence and strength of recommendations for diagnostic tests and strategies. BMJ.

[32] World Health Organization (2014). WHO indoor air quality guidelines: household fuel combustion.

[33] Office of Health Assessment and Translation (OHAT): Handbook for conducting a literature-based health assessment using OHAT approach for systematic review and evidence integration. National Toxicology Program; 2015.

[34] Hass U, Christiansen S, Axelstad M, Boberg J, Andersson A-M, Skakkebaek NE, Bay K, Holbech H, Kinnberg KL, Bjerregaard P (2012). Evaluation of 22 SIN list 2.0 substances according to the Danish proposal on criteria for endocrine disrupters.

[35] Woodruff TJ (2015). Making it real-the environmental burden of disease. What does it take to make people pay attention to the environment and health?. J Clin Endocrinol Metab.

[36] Lam J, Koustas E, Sutton P, Johnson PI, Atchley DS, Sen S, Robinson KA, Axelrad DA, Woodruff TJ (2014). The navigation guide - evidence-based medicine meets environmental health: integration of animal and human evidence for PFOA effects on fetal growth. Environ Health Perspect.

[37] Vandenberg LN, Agerstrand M, Beronius A, Beausoleil C, Bergman A, Bero LA, Bornehag CG, Boyer CS, Cooper GS, Cotgreave I, Gee D, Grandjean P, Guyton KZ, Hass U, Heindel JJ, Jobling S, Kidd KA, Kortenkamp A, Macleod MR, Martin OV, Norinder U, Scheringer M, Thayer KA, Toppari J, Whaley P, Woodruff TJ, Ruden C (2016). A proposed framework for the systematic review and integrated assessment (SYRINA) of endocrine disrupting chemicals. Environ Health.

[38] Trasande L, Zoeller RT, Hass U, Kortenkamp A, Grandjean P, Myers JP, DiGangi J, Bellanger M, Hauser R, Legler J, Skakkebaek NE, Heindel JJ (2015). Estimating burden and disease costs of exposure to endocrine-disrupting chemicals in the European union. J Clin Endocrinol Metab.

[39] Attina TM, Hauser R, Sathyanarayana S, Hunt PA, Bourguignon JP, Myers JP, DiGangi J, Zoeller RT, Trasande L (2016). Exposure to endocrine-disrupting chemicals in the USA: a population-based disease burden and cost analysis. Lancet Diabetes Endocrinol.

[40] Rice DP, Hodgson TA, Sinsheimer P, Browner W, Kopstein AN (1986). The economic costs of the health effects of smoking, 1984. The Milbank Quarterly.

[41] Hodgson TA (1981). Social and economic implications of cancer in the United States. Ann N Y Acad Sci.

[42] Weiss KB, Sullivan SD, Lyttle CS (2000). Trends in the cost of illness for asthma in the United States, 1985-1994. J Allergy Clin Immunol.

[43] Hodgson TA, Meiners MR: Cost-of-illness methodology: a guide to current practices and procedures. The Milbank Memorial Fund quarterly health and Society 1982, 60:429–462. 6923138

[44] Salkever DS (1995). Updated estimates of earnings benefits from reduced exposure of children to environmental lead. Environ Res.

[45] Schwartz J (1994). Societal benefits of reducing lead exposure. Environ Res.

[46] Hanushek EA, Woessmann L (2008). The role of cognitive skills in economic development. J Econ Lit.

[47] Rollins NC, Bhandari N, Hajeebhoy N, Horton S, Lutter CK, Martines JC, Piwoz EG, Richter LM, Victora CG (2016). Lancet breastfeeding series G: why invest, and what it will take to improve breastfeeding practices?. Lancet.

[48] Rabl A, Spadaro JV, Holland M (2014). How much is clean air worth? Calculating the benefits of pollution control.

[49] van Grinsven HJ, Rabl A, de Kok TM (2010). Estimation of incidence and social cost of colon cancer due to nitrate in drinking water in the EU: a tentative cost-benefit assessment. Environ Health.

[50] Desaigues B, Ami D, Bartczak A, Braun-Kohlová M, Chilton S, Czajkowski M, Farreras V, Hunt A, Hutchison M, Jeanrenaud C, Kaderjak P, Máca V, Markiewicz O, Markowska A, Metcalf S, Navrud S, Nielsen JS, Ortiz R, Pellegrini S, Rabl A, Riera R, Scasny M, Stoeckel ME, Szántó R, Urban J (2007). Economic valuation of air pollution mortality: a 9-country contingent valuation survey of value of a life year. Ecol Indic.

[51] World Health Organization (2015). The Minsk declaration: the life-course approach in the context of health.

[52] World Health Organization (2016). Ambient air pollution: a global assessment of exposure and burden of disease.

[53] Attina TM, Trasande L (2013). Economic costs of childhood lead exposure in low- and middle-income countries. Environ Health Perspect.

[54] Tsai PL, Hatfield TH (2011). Global benefits from the phaseout of leaded fuel. J Environ Health.

[55] Karagas MR, Choi AL, Oken E, Horvat M, Schoeny R, Kamai E, Cowell W, Grandjean P, Korrick S (2012). Evidence on the human health effects of low-level methylmercury exposure. Environ Health Perspect.

[56] Bellanger M, Pichery C, Aerts D, Berglund M, Castano A, Cejchanova M, Crettaz P, Davidson F, Esteban M, Fischer ME, Gurzau AE, Halzlova K, Katsonouri A, Knudsen LE, Kolossa-Gehring M, Koppen G, Ligocka D, Miklavcic A, Reis MF, Rudnai P, Tratnik JS, Weihe P, Budtz-Jorgensen E, Grandjean P (2013). Economic benefits of methylmercury exposure control in Europe: monetary value of neurotoxicity prevention. Environ Health.

[57] Trasande L, Liu Y (2011). Reducing the staggering costs of environmental disease in children, estimated at $76.6 billion in 2008. Health Aff.

[58] Grandjean P, Pichery C, Bellanger M, Budtz-Jorgensen E (2012). Calculation of mercury's effects on neurodevelopment. Environ Health Perspect.

[59] Bender M: Assessing hair mercury levels of women of childbearing age in 9 countries. Brussels: Zero Mercury Working Group 2013: 28.

[60] Trasande L, DiGangi J, Evers DC, Petrlik J, Buck DG, Samanek J, Beeler B, Turnquist MA, Regan K (2016). Economic implications of mercury exposure in the context of the global mercury treaty: hair mercury levels and estimated lost economic productivity in selected developing countries. J Environ Manag.

[61] Sheehan MC, Burke TA, Navas-Acien A, Breysse PN, McGready J, Fox MA (2014). Global methylmercury exposure from seafood consumption and risk of developmental neurotoxicity: a systematic review. Bull World Health Organ.

[62] Chen A, Yolton K, Rauch SA, Webster GM, Hornung R, Sjodin A, Dietrich KN, Lanphear BP (2014). Prenatal Polybrominated Diphenyl ether exposures and neurodevelopment in U.S. children through 5 years of age: the HOME study. Environ Health Perspect.

[63] Eskenazi B, Chevrier J, Rauch SA, Kogut K, Harley KG, Johnson C, Trujillo C, Sjodin A, Bradman A (2013). Utero and childhood Polybrominated Diphenyl ether (PBDE) exposures and neurodevelopment in the CHAMACOS study. Environ Health Perspect.

[64] Herbstman JB, Sjodin A, Kurzon M, Lederman SA, Jones RS, Rauh V, Needham LL, Tang D, Niedzwiecki M, Wang RY, Perera F (2010). Prenatal exposure to PBDEs and neurodevelopment. Environ Health Perspect.

[65] Engel SM, Wetmur J, Chen J, Zhu C, Barr DB, Canfield RL, Wolff MS (2011). Prenatal exposure to organophosphates, Paraoxonase 1, and cognitive development in childhood. Environ Health Perspect.

[66] Bouchard MF, Chevrier J, Harley KG, Kogut K, Vedar M, Calderon N, Trujillo C, Johnson C, Bradman A, Barr DB, Eskenazi B (2011). Prenatal exposure to organophosphate pesticides and IQ in 7-year old children. Environ Health Perspect.

[67] Rauh V, Arunajadai S, Horton M, Perera F, Hoepner L, Barr DB, Whyatt R (2011). 7-year neurodevelopmental scores and prenatal exposure to Chlorpyrifos, a common agricultural pesticide. Environ Health Perspect.

[68] Zoeller RT, Brown TR, Doan LL, Gore AC, Skakkebaek NE, Soto AM, Woodruff TJ, Vom Saal FS (2012). Endocrine-disrupting chemicals and public health protection: a statement of principles from the Endocrine Society. Endocrinology.

[69] Bellanger M, Demeneix B, Grandjean P, Zoeller RT, Trasande L (2015). Neurobehavioral deficits, diseases, and associated costs of exposure to endocrine-disrupting chemicals in the European Union. J Clin Endocrinol Metab.

[70] Ravenscroft P, Brammer, H., Richards, K.: Arsenic pollution: a global synthesis. Chichester: Wiley-Blackwell; 2009.

[71] Fleischer NL, Merialdi M, van Donkelaar A, Vadillo-Ortega F, Martin RV, Betran AP, Souza JP (2014). Outdoor air pollution, preterm birth, and low birth weight: analysis of the world health organization global survey on maternal and perinatal health. Environ Health Perspect.

[72] Guxens M, Ghassabian A, Gong T, Garcia-Esteban R, Porta D, Giorgis-Allemand L, Almqvist C, Aranbarri A, Beelen R, Badaloni C, Cesaroni G, de Nazelle A, Estarlich M, Forastiere F, Forns J, Gehring U, Ibarluzea J, Jaddoe VW, Korek M, Lichtenstein P, Nieuwenhuijsen MJ, Rebagliato M, Slama R, Tiemeier H, Verhulst FC, Volk HE, Pershagen G, Brunekreef B, Sunyer J (2016). Air pollution exposure during pregnancy and childhood autistic traits in four European population-based cohort studies: the ESCAPE project. Environ Health Perspect.

[73] Sunyer J, Esnaola M, Alvarez-Pedrerol M, Forns J, Rivas I, Lopez-Vicente M, Suades-Gonzalez E, Foraster M, Garcia-Esteban R, Basagana X, Viana M, Cirach M, Moreno T, Alastuey A, Sebastian-Galles N, Nieuwenhuijsen M, Querol X (2015). Association between traffic-related air pollution in schools and cognitive development in primary school children: a prospective cohort study. PLoS Med.

[74] Woodruff TJ, Parker JD, Darrow LA, Slama R, Bell ML, Choi H, Glinianaia S, Hoggatt KJ, Karr CJ, Lobdell DT (2009). Methodological issues in studies of air pollution and reproductive health. Environ Res.

[75] U.S. Environmental Protection Agency: Integrated science assessment of ozone and related photochemical oxidants (final report). U.S. EPA; 2013.

[76] Institute of Medicine (2007). Preterm birth: causes, consequences, and prevention.

[77] Suades-Gonzalez E, Gascon M, Guxens M, Sunyer J (2015). Air pollution and neuropsychological development: a review of the latest evidence. Endocrinology.

[78] Driscoll T, Takala J, Steenland K, Corvalan C, Fingerhut M (2005). Review of estimates of the global burden of injury and illness due to occupational exposures. Am J Ind Med.

[79] Pasetto R, Comba P, Marconi A (2005). Mesothelioma associated with environmental exposures. Med Lav.

[80] Ramazzini C (2016). The global health dimensions of asbestos and asbestos-related diseases. Med Lav.

[81] Trasande L, Zoeller RT, Hass U, Kortenkamp A, Grandjean P, Myers JP, DiGangi J, Hunt PM, Rudel R, Sathyanarayana S, Bellanger M, Hauser R, Legler J, Skakkebaek NE, Heindel JJ (2016). Burden of disease and costs of exposure to endocrine disrupting chemicals in the European Union: an updated analysis. Andrology.

[82] Bartlett ES, Trasande L (2014). Economic impacts of environmentally attributable childhood health outcomes in the European Union. Eur J Pub Health.

[83] Trasande L, Malecha P, Attina TM (2016). Particulate matter exposure and preterm birth: estimates of U.S. attributable burden and economic costs. Environ Health Perspect.

[84] Hauser R, Skakkebaek NE, Hass U, Toppari J, Juul A, Andersson AM, Kortenkamp A, Heindel JJ, Trasande L (2015). Male reproductive disorders, diseases, and costs of exposure to endocrine-disrupting chemicals in the European union. J Clin Endocrinol Metab.

[85] Chatham-Stephens K, Caravanos J, Ericson B, Sunga-Amparo J, Susilorini B, Sharma P, Landrigan PJ, Fuller R (2013). Burden of disease from toxic waste sites in India, Indonesia, and the Philippines in 2010. Environ Health Perspect.

[86] Trasande L (2014). Further limiting bisphenol a in food uses could provide health and economic benefits. Health Aff.

[87] Hunt PA, Sathyanarayana S, Fowler PA, Trasande L (2016). Female reproductive disorders, diseases, and costs of exposure to endocrine disrupting Chemicals in the European Union. J Clin Endocrinol Metab.

[88] Woods B, Revill P, Sculpher M, Claxton K (2016). Country-level cost-effectiveness thresholds: initial estimates and the need for further research. Value Health.

[89] Bertram MY, Lauer JA, De Joncheere K, Edejer T, Hutubessy R, Kieny MP, Hill SR (2016). Cost-effectiveness thresholds: pros and cons. Bull World Health Organ.

[90] Janesick AS, Blumberg B (2016). Obesogens: an emerging threat to public health. Am J Obstetr Gynecol.

[91] Stieb DM, De Civita P, Johnson FR, Manary MP, Anis AH, Beveridge RC, Judek S (2002). Economic evaluation of the benefits of reducing acute cardiorespiratory morbidity associated with air pollution. Environ Health.

[92] Needham LL, Calafat AM, Barr DB (2008). Assessing developmental toxicant exposures via biomonitoring. Basic Clin Pharmacol Toxicol.

[93] Chatham-Stephens K, Caravanos J, Ericson B, Landrigan P, Fuller R (2014). The pediatric burden of disease from lead exposure at toxic waste sites in low and middle income countries. Environ Res.

[94] Blacksmith Institute (2012). The world’s worst pollution problems: assessing health risks at hazardous waste sites.

[95] Organization for Economic Cooperation and Development (2008). OECD environmental outlook to 2030. Chapter 18: Chemicals.

[96] Trasande L, Massey RI, DiGangi J, Geiser K, Olanipekun AI, Gallagher L (2011). How developing nations can protect children from hazardous chemical exposures while sustaining economic growth. Health Aff.

[97] Chanel O, Perez L, Kunzli N, Medina S, Aphekom G (2016). The hidden economic burden of air pollution-related morbidity: evidence from the Aphekom project. Eur J Health Econ.

[98] Trasande L, Malecha P, Attina TM. Particulate matter exposure and preterm birth: estimates of US attributable burden and economic costs. Environ Health Perspect. 2017;124:1913–18. 10.1289/ehp.1510810 PMC5132647 27022947

[99] Pascal M, Corso M, Chanel O, Declercq C, Badaloni C, Cesaroni G, Henschel S, Meister K, Haluza D, Martin-Olmedo P, Medina S, Aphekom G (2013). Assessing the public health impacts of urban air pollution in 25 European cities: results of the Aphekom project. Sci Total Environ.

[100] Organization for Economic Cooperation and Development (2014). The cost of air pollution: health impacts of road transport.

[101] Bierkens J, Buekers J, Van Holderbeke M, Torfs R (2012). Health impact assessment and monetary valuation of IQ loss in pre-school children due to lead exposure through locally produced food. Sci Total Environ.

[102] Perera F, Weiland K, Neidell M, Wang S (2014). Prenatal exposure to airborne polycyclic aromatic hydrocarbons and IQ: estimated benefit of pollution reduction. J Public Health Policy.

[103] Weiland K, Neidell M, Rauh V, Perera F (2011). Cost of developmental delay from prenatal exposure to airborne polycyclic aromatic hydrocarbons. J Health Care Poor Underserved.

[104] Brandt S, Perez L, Kunzli N, Lurmann F, Wilson J, Pastor M, McConnell R (2014). Cost of near-roadway and regional air pollution-attributable childhood asthma in Los Angeles County. J Allergy Clin Immunol.

[105] Brandt SJ, Perez L, Kunzli N, Lurmann F, McConnell R (2012). Costs of childhood asthma due to traffic-related pollution in two California communities. Eur Respir J.

