# Supplementary material for: Calculation of the disease burden associated with environmental chemical exposures: application of toxicological information in health economic estimation
Source: Environ Health. 2017 Dec 5;16:123. doi: 10.1186/s12940-017-0340-3 (PMC5715994; doi:10.1186/s12940-017-0340-3)
Supplement: Additional file 1: — Additional calculations of economic costs due to environmental disease burdens [101–105]. (DOC 39 kb) [file 12940_2017_340_MOESM1_ESM.doc]

**Supplementary file**

Additional file to “Calculation of the disease burden associated with environmental chemical exposure: Application of toxicological information in health economics estimates”

This supplementary table provides additional references to economic cost evaluations for particular adverse outcomes associated with individual environmental chemical exposures in various populations.

**Table S1**. Literature reports on economic costs for environmental risk factors

| Chemical risk factor | Adverse consequence | Context | Economic cost ($millions) |
| --- | --- | --- | --- |
| *Neurotoxicants* |  |  |  |
| Lead | Cognitive deficits | EU[101] | 26,600 |
| Polyaromatic hydrocarbons |  | New York City (US)[102] | 215 |
|  |  | New York City (US)[103] | 168 |
| *Air pollution* |  |  |  |
| Near road traffic related pollution | Childhood asthma | EU cities[97] | 152 |
|  | Heart disease in elderly |  | 388 |
| Ozone | Childhood asthma | Los Angeles county (US)[104] | 441 |
| Nitrogen oxides |  |  | 202 |
| Traffic related |  | Two California communities (US)[105] | 18 |
| Ozone (5 µg/m^3^) | Respiratory mortality | EU[99] | 826 |
| Particulate matter (above 10μg/m^3^) | Cardiovascular mortality |  | 37,187 |
| *Other chemicals* |  |  |  |
| Aldrin | Liver cancer and toxicity | Communities near hazardous waste sites in India, Indonesia and Philippines[85] | 51.2 |
| Asbestos | Lung cancer |  | 260 |
| Cadmium |  |  | 0.75 |
| Chromium (VI) |  |  | 14,900 |
| Dichlorodiphenyl-trichloroethane (DDT) | Liver cancer and toxicity |  | 1.10 |
| Lindane |  |  | 4.95 |
| Inorganic mercury | Renal toxicity |  | 4.15 |

All estimates are given in $2010, a 1.33 exchange rate for € /$ is used, and for estimates prior to 2010, inflated adjustments are made. No overall estimate is provided in this table, since the cost estimates come from different sources and estimation methods.
